# Traumatic prolapse of the globe into the anterior cranial fossa: a case report

**DOI:** 10.1186/s12886-020-01403-2

**Published:** 2020-04-03

**Authors:** Hui Liu, Shengli Hu, Wei Qin

**Affiliations:** 1 Department of Ophthalmology, Chengdu Aier Eye Hospital, Chengdu, China; 2 Department of Ophthalmology, Southwest Hospital, Third Military Medical University, Chongqing, China; 3 Department of Neurosurgery, Southwest Hospital, Third Military Medical University, Chongqing, China; 4 Department of Ophthalmology, Chungking General Hospital, Chongqing, China

**Keywords:** Traumatic dislocation of the globe, Extraocular muscle injury, Blow-out fractures, Orbitocranial trauma

## Abstract

**Background:**

Orbital fracture associated with traumatic intracranial prolapse of the eyeball is rare. In all previously reported cases, vision was severely impaired with no light perception. Herein, we report a case of traumatic prolapse of the globe into the anterior cranial fossa, in which the patient’s vision was preserved by early repositioning.

**Case presentation:**

The present case report focused on a man hit by a steel pipe, leading to prolapse of the globe of the right eye into the anterior cranial fossa through fractures in the superior orbit roof, accompanied by cerebral contusion. The eyeball was immediately repositioned into the orbital cavity, along which the wound tract was debrided and the skull base was repaired. The patient underwent a follow-up period of 12 months, during which the visual acuity increased to 12/20 without any intracranial infections. However, the patient’s ptosis persisted and was associated with complete loss of supraduction.

**Conclusions:**

In this case, early diagnosis and proper globe repositioning with reconstruction of the orbital roof could allow recovery of vision, as well as prevention of intracranial infection.

## Background

Dislocation of the globe mainly occurs inferiorly into the maxillary sinus or medially into the ethmoid sinus following significant blunt facial trauma [1]. Only three cases of eyeball extrusion into the cranial cavity have been reported to date [2]. Although relocation of orbital contents prevented disfigurement in the previously reported cases, it was believed that visual prognosis was poor in the extruded eyeball due to either mechanical or vascular insult [2, 3]. However, the present case not only showed the cosmetic restoration but also the improvement of postoperative vision. This report highlights the importance of early surgical intervention and the technique of globe repositioning in the treatment of traumatic intracranial prolapse of eyeball.

## Case presentation

**Fig. 1:**
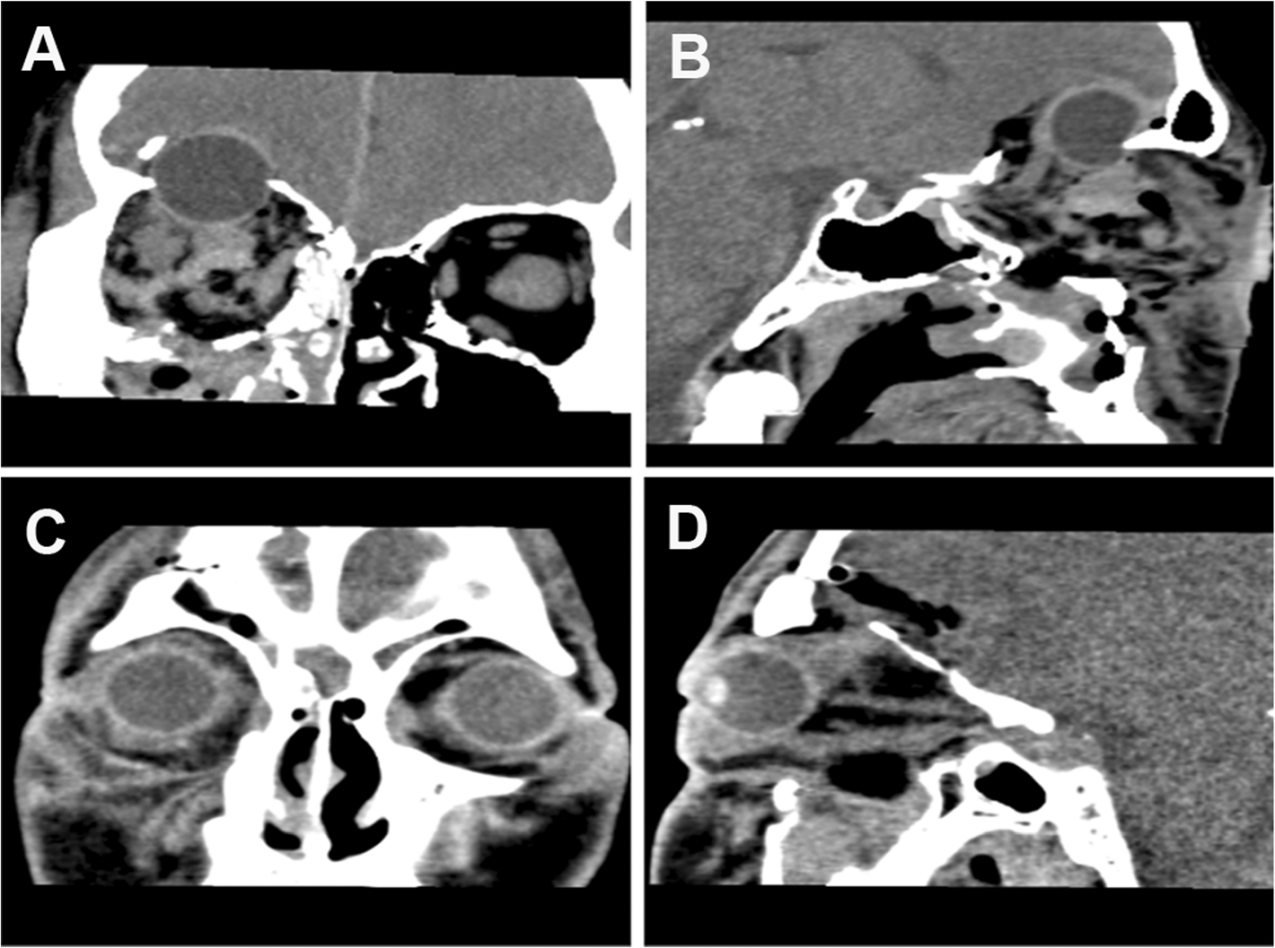
**a** Coronal CT scan showing large fractures of the right orbital roof and incomplete dislocation of the right globe into the anterior cranial fossa. **b** Sagittal CT scan showing that no fractures were noted in the optic canal, but that the optic nerve appeared kinked. **c** Coronal CT scan showing that the dislocated globe was repositioned to its correct anatomical position on postoperative day 1. **d** Sagittal CT scan showing the continuity of the optic nerve on postoperative day 1

A 32-year-old man was brought to our emergency department, having sustained extensive trauma of the right eye and orbit after being hit by a steel pipe for 3 h. On physical examination, the patient exhibited marked periorbital swelling unilaterally and a full-thickness, oblique laceration on the right lower eyelid. Upon retraction of the right eyelid, marked conjunctival edema and hemorrhage were noted, and no globe was found on visual inspection. The patient had a Glasgow coma score of 15 with no evidence of any neurological dysfunction. No cerebrospinal fluid (CSF) leakage was noted. A computed tomography (CT) scan revealed that the slightly compressed, deformed globe had extruded into the anterior cranial fossa through large fractures in the orbital roof, accompanied by cerebral contusion (Fig. 1a, b). No fractures were noted in the optic canal, but the optic nerve appeared kinked (Fig. 1b).

Following the diagnosis, the patient was immediately transported to the operating room for globe repositioning and repair of the skull base under general anesthesia. The dura was opened, and the eyeball was found situated at the inferior surface of the frontal lobe. After removal of the thin bone fragments in the skull base, the neurosurgeon made two attempts to push the globe back into the orbit cavity. However, the eyeball could not be repositioned and slowly retruded into the cranial cavity.

Next, an orbital exploration was performed using a transconjunctival approach. After dissecting the orbital rim through a pre-existing tissue laceration, the globe was noted to have been displaced superiorly with exposure of the sclera. Forceps were used to grasp the residual Tenon’s capsule attached to the eyeball to guide the globe inferiorly, from the orbital roof into the orbital cavity, and then to rotate the eyeball with the cornea facing forward. Care was taken not to abruptly retract the globe in order to avoid incurring any additional injury to the optic nerve.

The status of the extraocular muscles was next assessed. The medial and inferior rectus muscles were torn, nearly from the middle of the muscle belly. However, we were unable to find the distal sections of the medial and inferior rectus muscles. On gross examination, we noted no signs of eyeball rupture and the pupil appeared almost normal with slight hyphema.

After removing the globe, CSF leakage was noted. Debridement and irrigation were performed at the wound tract. Some bone fragments were placed back at the bone-defective region. The dura was sutured as naturally as possible. Periosteal flaps with pedicles were then transferred to the anterior cranial fossa to cover the dural defect. Biomedical fibrin glue was injected into the flaps to enable adherence. Next, an artificial duramater was used to reinforce the repair. Finally, the wound was closed and a dressing was applied.

The patient received intravenous antibiotics and a high dose of corticoids. Tropical steroid drops were used as anti-inflammatories. One day after the surgery, the patient showed signs of ptosis of the right eye. On lifting the upper eyelid using a cotton swab, he reported seeing signs of hand movement in front of his right eye. On a bedside slit-lamp examination, his cornea was clear, blood clots were present in the anterior chamber with a nonreactive pupil (4.0 mm), and the intraocular pressure was 11 mmHg. A CT scan was performed on the first postoperative day, revealing the correct anatomic positioning (Fig. 1c). The optic nerve was seen to be continuous (Fig. 1d). One week postoperatively, the blood clots in the anterior chamber were partially resolved with an increase in visual acuity of 2/20, despite continued ptosis. Three months following surgery, there were no abnormal findings in the anterior or posterior segments, except for slight symblepharon and a nonreactive pupil. The patient has undergone 12 months of follow-up during which his visual acuity increased to 12/20. The globe had slightly deviated to the inferolateral side from the primary position, but infraduction, abduction, and adduction were proven to be possible as shown in the video (Additional file 1). However, the ptosis persisted and was associated with complete loss of supraduction. The patient gradually developed moderate enophthalmos.

## Discussion and Conclusion

The management of cranial-orbital injuries is currently a challenge for ophthalmologists, neurosurgeons, and neuroradiologists and requires innovative planning. Previous studies have shown that the dislocation of the globe into either the paranasal sinuses or nasal cavities [1]. In contrast, the patient in this case was suffering from a traumatic globe extruded into the anterior cranial fossa, which has been rarely reported in the literature [2].

The most widely accepted mechanism for orbital blowout-type fractures is an associated increase in intraorbital pressure, when blunt forces to either the orbital rim or the globe itself push the orbital tissue posteriorly. The increased intraorbital pressure causes the orbital bones to break or buckle at their weakest point. This weakest point is most commonly the posterior aspect of the orbital floor, medial to the infraorbital canal and the lamina papyracea of the ethmoid bone [4]. However, blowout fractures of the orbital roof are rarely seen. In contrast, the globe itself is suspended and supported within the orbit by a combination of muscular and bulbar fascial sheaths, extraocular muscles, ligamentous tissue, and orbital fat [5]. In addition to suspending the globe within the orbit, the periorbital tissues act as soft tissue padding and are able to dissipate considerable energy. Thus, blunt trauma to the ocular region can produce significant contusions and fractures of the orbit, while causing little or no damage to the globe itself [6].

Surgical approaches are tailored to intracranial- and intraorbital-associated injuries and complications. Vahdati et al. [7] reported that the eyeball was retrieved by traction through the conjunctival sac, whereas Gollapudi et al. [2] performed craniotomy and excised the orbital roof on either side of the fracture to replace the globe into the orbit. In the present case, images from coronal and sagittal CT scans offered additional preoperative details, which showed that the eyeball was buckled by the fractures of the orbital roof, accompanied by cerebral contusion. Thus, we chose to approach the eyeball through a right fronto-temporal craniotomy as the first step rather than aggravate the compression of the eyeball by retrieving it directly through the conjunctival sac. In order to avoid secondary injury, we only removed the thin bone fragments rather than excise the orbital roof. When the globe encountered orbital resistance during repositioning, we changed the approach by performing orbital exploration through the conjunctival sac. Subsequently, globe repositioning became much safer as the globe could be directly visualize.

Moreover, the purpose of treating a transorbital penetrating injury is to save the patient’s life by controlling persistent bleeding and intracranial hypertension, preventing infection through debridement of all contaminated and necrotic tissues, preserving as much nerve tissue as possible, and restoring anatomical structures through the accurate closure of the dura and scalp [8]. It has been previously reported that mortality rates were high due to infections and lack of optimal antimicrobial therapy [9]. Therefore, after removing the globe, we chose to thoroughly visually debride and irrigate the wound tract at its intracranial level. Since bone reconstruction can be associated with a risk of infection, a multi-layered closure was performed to repair the orbital roof in this case, which had proved to be effective in preventing leakage of cerebrospinal fluid and subsequent meningitis [10].

Most researchers have agreed that the dislocated globe should be replaced into the orbital cavity as soon as possible [4, 11, 12]. Avulsion of the optic nerve and its blood supply is the most common mechanism producing total loss of vision in an intact, severely displaced globe [11]. Treatment delays may increase the risk of complications such as edema and strain on adnexal structures, especially in the optic nerve and central retinal artery, and this risk increases with time. A twisted or stretched retinal artery may damage the blood supply of the optic nerve, increasing the probability of irreversible vision loss [3, 4, 11]. Besides, the variability of visual outcomes also depends on the degree to which the optic nerve is distorted [13]. However, the condition of the optic nerve was difficult to ascertain preoperatively [2, 7], thus we used sagittal CT scan to show the continuity of the optic nerve (Fig. 1b). Recovery of visual acuity was only found in approximately 1/3 of cases with dislocation of the eyeball into the maxillary sinus, which had the globe repositioned on the same day as the trauma [3]. Consistent with the surgery time window for dislocated eyes, in our patient, the globe extruding into the anterior cranial fossa was also repositioned within 24 h and visual acuity was regained. Compared with prior reports, the present case was the earliest to receive repositioning surgery, which was associated with good visual prognosis.

Early diagnosis and immediate globe repositioning are important for a patient’s visual recovery if the optic nerve remains viable. Repairing the orbital roof may not only prevent CSF leakage, but also prevent intracranial infection.

## Supplementary information

**Additional file 1.** Position of the eye and its movement after surgery.

## Data Availability

The datasets from the current study can be obtained on request from the corresponding author.

## Abbreviations

CSF: Cerebrospinal fluid
CT: Computed tomography
